# Key Players in HIV-1 Transcriptional Regulation: Targets for a Functional Cure

**DOI:** 10.3390/v12050529

**Published:** 2020-05-11

**Authors:** Luisa Mori, Susana T. Valente

**Affiliations:** Department of Immunology and Microbiology, The Scripps Research Institute, 130 Scripps Way, Jupiter, FL 33458, USA; lmori@scripps.edu

**Keywords:** HIV-1 transcription, epigenetics, therapeutic approaches

## Abstract

HIV-1 establishes a life-long infection when proviral DNA integrates into the host genome. The provirus can then either actively transcribe RNA or enter a latent state, without viral production. The switch between these two states is governed in great part by the viral protein, Tat, which promotes RNA transcript elongation. Latency is also influenced by the availability of host transcription factors, integration site, and the surrounding chromatin environment. The latent reservoir is established in the first few days of infection and serves as the source of viral rebound upon treatment interruption. Despite effective suppression of HIV-1 replication by antiretroviral therapy (ART), to below the detection limit, ART is ineffective at reducing the latent reservoir size. Elimination of this reservoir has become a major goal of the HIV-1 cure field. However, aside from the ideal total HIV-1 eradication from the host genome, an HIV-1 remission or functional cure is probably more realistic. The “block-and-lock” approach aims at the transcriptional silencing of the viral reservoir, to render suppressed HIV-1 promoters extremely difficult to reactivate from latency. There are unfortunately no clinically available HIV-1 specific transcriptional inhibitors. Understanding the mechanisms that regulate latency is expected to provide novel targets to be explored in cure approaches.

## 1. Introduction

Despite the global success of anti-retroviral therapy (ART), the persistence of a pool of latently infected cells, termed the viral reservoir, represents one of the major barriers to cure HIV-1. This viral reservoir consists of cells harboring integrated, transcriptionally silent proviruses that, upon treatment interruption, are capable of producing fully infectious virions to reignite rebound viremia [1,2,3]. A sterilizing cure for HIV-1 is, by definition, the total eradication of the viral reservoir. Despite tremendous efforts in this direction, we are still far from this goal and additional efforts have been focused on achieving a functional cure, whereby durable suppression of HIV-1 expression can be achieved. A functional cure for HIV-1 would entail no viral replication in the absence of ART, no risk of onward transmission, no ongoing virological damage; in sum, detectable viral DNA but no evidence of virus production from competent integrated viruses [4,5,6]. ART regimens are extremely successful at reducing the circulating viral loads in the plasma of infected individuals to below the limit of detection, thus, preventing transmission and disease progression [7]. Infected individuals must however remain on ART for the rest of their lives, because as soon as treatment is interrupted, viral loads rebound back to pre-ART levels. Unfortunately, there are no clinically approved HIV-1 transcriptional inhibitors that could block ongoing transcriptional events in infected cells. Significant progress has been made in the past few decades towards understanding underlying mechanisms that regulate HIV-1 transcription and latency; however, much remains to be understood. Here, we review recent advances in the field of HIV-1 transcription and discuss novel studies that identify potential drug targets for the development of novel therapeutic strategies towards a functional HIV-1 cure.

HIV-1 was shown to preferentially integrate into the gene bodies of actively transcribed regions of the host chromatin, in studies of cell lines, primary CD4+T cells, as well as in patient-derived CD4+T cells [8,9,10]. Once integrated into the host chromosome, virus transcriptional regulation is dependent on the complex interplay of a plethora of host and viral factors, which will be the topic of this review. Cells that express high levels of viral RNA, proteins or virions are selected against, through cytopathic effects or immune-mediated cytotoxic killing. Latently infected cells, on the other hand, have little to no expression of viral RNA or proteins, and can survive by flying under the immune system’s radar. Unlike some other viruses that undergo latent stages, such as herpesviruses, HIV-1 does not have a defined latency program [11]. HIV-1 entry into latency is a complex and incompletely understood pathway. Factors implicated in driving the virus into latency include the depletion of the viral transactivator of transcription protein (Tat) [12,13,14], a reduction in the availability of cellular transcription factors (TFs) [15,16,17,18], an accumulation of epigenetic silencing marks surrounding the HIV-1 promoter [19], and the activity of additional chromatin regulators [20,21]. We will discuss these factors and their interconnectivity in this review.

Viruses have evolved strategies to precisely fine tune host regulatory transcription programs, and HIV-1 is no exception. Delivery of RNAPII to latent HIV-1 promoters, which are generally buried in repressive nucleosomes, involves the collective action of several sequence-specific DNA binding TFs. These allow recruitment of the transcriptional machinery and initiation of mRNA synthesis. Additional TFs are recruited to induce productive elongation, splicing, and polyadenylation to generate fully processed, mature HIV-1 mRNAs. A plethora of TFs have been shown to bind the HIV-1 genome in vitro and regulate proviral transcription. Some factors are critical for HIV transcriptional control, for example nuclear factor (NF)-κB, nuclear factor for activated T cells (NFAT), specificity protein 1 (Sp1), and TATA-binding protein (TBP) [22]. Factors such as AP-1, play important accessory roles in transcription modulation; their deletion, while negatively impacting transcription, does not abrogate latency establishment or reactivation [23]. Additional TFs which bind the HIV-1 promoter, such as lymphocyte enhancer (LEF-1) and Yin Yan 1 (YY1), serve cooperative or modulatory functions to fine-tune viral expression, and their over-expression or deletion has been shown to directly impact HIV expression [19,24,25].

HIV-1 enhances its own transcription and modulates the expression of cellular genes, and this is mainly due to the activity of the viral protein Tat [26,27,28]. Tat serves as a molecular switch between latency and active transcription [29]. This is primarily achieved through recruitment of the positive elongation factor (P-TEFb), relieving promoter-proximal stalling through phosphorylation of RNAPII, negative elongation factor (NELF) and DRB sensitivity inducing factor (DSIF), described in Section 5 [30,31,32,33]. Whether Tat exclusively modulates elongation, or both initiation and elongation, has been debated [34]. Some studies showed no impact of Tat on initiation, with short transcripts being transcribed equally well with or without Tat; while RNAs longer than ~59 nt dramatically increased in the presence of Tat [35,36,37]. Others have shown that Tat promotes up to 15-fold increase in the production of shorter 24 nt transcripts from the transcription start site (TSS), before the TAR RNA has been fully transcribed [38]. Subsequent work has supported Tat’s role in initiation [34]. For instance, one study has shown Tat recruitment of components of the pre-initiation complex (PIC), including the mediator complex components and the TATA-binding protein (TBP), independent of the TBP-associated factors (TAFs), in addition to P-TEFb [39]. In this study, artificial tethering of Tat or components of P-TEFb to DNA resulted in the same recruitment of TBP to the LTR, in the absence of TAFs. Thus, suggesting that Tat binding to the TAR RNA, recruits P-TEFb and additional components of the PIC, such as Mediator and TBP, to promote initiation, in addition to elongation. This study corroborates an earlier observation of P-TEFb present in the PIC on the HIV-1 promoter [40]. Further investigation is needed to fully understand the mechanism by which P-TEFb recruits TBP without TAFs. It has also been demonstrated that Tat recruits histone acetyl transferases (HATs), including p300/CEBP, PCAF, and hGCN5 to the promoter, which are all activators of HIV-1 transcription and could promote initiation [41,42]. It is thus very likely that both transcriptional initiation and elongation are enhanced through the positive-feedback loop established by Tat. Low levels of nascent TAR RNA detected during latency [43] brings Tat in close proximity to the promoter, recruiting P-TEFb and facilitating elongation and subsequent rounds of initiation, through an enhanced PIC assembly and HAT recruitment. Discrepancies in these early studies might be attributed to differences in the experimental systems used. For example, in vitro transcription experiments using naked DNA templates might not fully recapitulate the regulation of transcription in cells. Furthermore, in order to fully appreciate the role of Tat in transcription, multiple rounds of transcription need to be sustained, to allow a positive-feedback loop to be established. Altogether, the role of Tat in both productive elongation and initiation together potentiate the robust increase in full-length HIV mRNA transcripts, upon reactivation. Additionally, Tat associates with other TFs to modulate the expression of host genes, to promote a permissive environment for HIV-1 replication, and evasion of immune responses [44]. Given Tat’s essential role in transcription, conditions that restrict its levels, such as reduced initiation of transcription, will in turn affect the positive feedback loop established by Tat. When the levels of Tat drop below a certain threshold, transcriptional elongation is drastically inhibited, driving the virus into latency [45,46,47].

Understanding the mechanisms by which HIV-1 hijacks cellular TFs to promote its own expression of its genome is a significant area of active research, as it is expected to reveal novel potential targets for drug development towards a cure.

## 2. HIV-1 Promoter Structure

**Figure 1:**
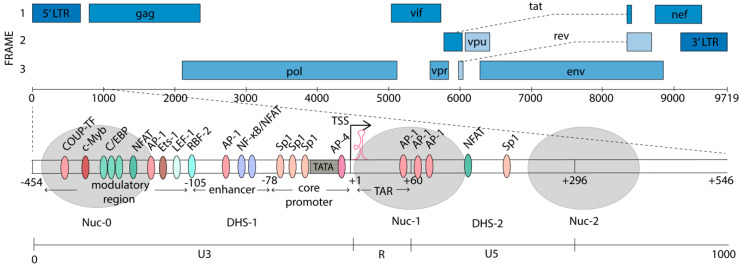
Structure of the HIV-1 promoter. The integrated HIV-1 genome is 9719 nucleotides in length. It includes two copies of the LTR which consist of a U3, R and U5 region, shown in the bottom panel. The genome encodes 9 genes which are shown in the top panel. The 5’LTR serves as the promoter for HIV-1 and is divided into the modulatory, enhancer, core promoter and TAR regions (shown enlarged in the middle panel). The binding sites for some of the ~50 TFs known to interact with the HIV-1 promoter are shown by colored ovals. The TATA box is shown by the grey rectangle, which serves as the binding site for the TATA binding protein (TBP) as part of the general transcription factor TFIID, to allow formation of the pre-initiation complex, just before the transcription start site (TSS), marked by the black arrow. The positions of the three nucleosomes, (Nuc-0, -1, and -2) on the HIV-1 5’LTR are marked by the grey shaded ovals. The DNase hypersensitivity regions (DHS-1 and -2) are the nucleosome-free regions on the promoter.

The HIV-1 genome is flanked by two long terminal repeats (LTRs), which are structurally divided into U3, R, and U5 regions (Figure 1). The 5’ LTR serves as the dominant promoter, and transcription from the 3’ LTR is suppressed in the presence of a functional 5’ LTR [48]. The antisense transcript (AST) has been identified as an antisense transcriptional product driven by the 3’LTR, at ~100–2500-fold lower abundance than the sense transcripts [49,50]. The AST has been suggested to promote HIV-1 latency, by creating a repressive epigenetic environment on the HIV-1 promoter [49]. The 5’LTR is a strong promoter with over fifty different confirmed or predicted TF binding sites [51]. Many of these binding sites and the roles they play have been identified in cell line models of latency and the function of many of these factors is yet to be established in primary cells from HIV-1-infected individuals. The balance between binding of transcriptional activators and repressors on the HIV-1 promoter controls transcriptional activity and context-dependent expression. Additionally, once integrated, the HIV-1 genome adopts a unique chromatin architecture, where nucleosomes are specifically deposited on either side of the TSS to modulate HIV-1 gene expression [52]. Nucleosome-0 (Nuc-0) is located at ~400 nt upstream from the TSS, in the modulatory region of the promoter. Nuc-1, analogous to the +1 nucleosome in cellular genes, is positioned immediately downstream from the TSS [53,54]. The position of Nuc-1 occludes the transcriptional machinery from elongating and is associated with HIV-1 latency [52]. Upon cellular activation, Nuc-1 is specifically and rapidly remodeled to enhance transcription. The positions of the nucleosomes on the HIV-1 promoter are shown in Figure 1.

The HIV-1 promoter can be divided into four main regions, relative to the TSS at +1: a modulatory region (−455 to −104), an enhancer element (−109 to −79), the core promoter (−78 to −1), and the TAR region (+1 to +60) [51]. The region downstream of the TSS (+60 to +278) also binds TFs which play important roles in the transcriptional modulation.

The modulatory region that spans from −454 to −104 nt in the U3 region of the 5’LTR was initially thought to play a repressive role in transcription, but was later shown to contain binding sites for a multitude of both activating and repressive TFs, thus, playing more of a modulatory role [51]. For example, the CCAAT/enhancer binding proteins (C/EBP) has been shown to activate HIV-1 transcription in monocytes and macrophages [55], while USF/MLTF was shown to repress HIV transcription in HeLa cells [56]. Three binding sites for C/EBP, a LEF-1 site, and binding sites for NFAT, c-Myb, Ets-1, and AP-1, in addition to a number of other factors are found in this region [51,54]. The 5’ portion of the modulatory region is encompassed by Nuc-0 and the remaining portion is part of the DNase hypersensitivity (DHS)-1 region, leaving the DNA more accessible to TFs and transcription machinery.

The enhancer element (−137 to −17) was discovered by its ability to activate transcription from a heterologous promoter, irrespective of its orientation or distance from the promoter in infected cell lines [57]. It is located just upstream of the HIV-1 core promoter and contains tandem repeats of NF-κB consensus binding sites and related factors [58]. NF-κB is tightly controlled through interaction of its p65 subunit (also known as RelA) with the inhibitory protein IκB. IκB binds p65, which is part of a heterodimer with p50, in the cytoplasm, masking its nuclear localization signal [59]. Upon cellular activation and cytokine signaling, mitogen-activated IκB-kinase (IKK) phosphorylates IκB, resulting in IκB ubiquitination and degradation [60]. The p65/p50 heterodimer is then released and, once in the nucleus, binds its cognate sites on the HIV-1 promoter, to recruit components of the PIC [61]. NF-κB cooperates with Sp1 as well as other TFs that are highly prevalent in T cells (e.g., LEF-1 and Ets-1) to activate HIV-1 transcription [22]. The two NF-κB sites can also be occupied by NFAT and these are thought to be mutually exclusive [62]. NFAT, has been shown to play a crucial role in HIV-1 reactivation in primary memory CD4^+^T cells [63,64]. Upon T cell activation, Ca^2+^/calcineurin signaling results in the dephosphorylation of NFAT by the phosphatase calcineurin, triggering NFAT translocation to the nucleus [65]. NFAT thus provides another pathway for HIV upregulation, which can be achieved by T-cell activation [66]. Additionally, HSP70 binding protein 1 (HspBP1) also binds the enhancer NF-κB sites, thus blocking the binding of the p65/p50 heterodimer and suppressing the HIV-1 transcription [67]. Occupation of the NF-κB sites with p50 homodimers promotes latency by recruiting negative transcriptional regulators and blocking binding of positive regulators [18,68]. Upstream of the NF-κB sites is an AP-1 element that also participates in latency establishment [23]. In subtype B viruses this is a 4-nt AP-1 sequence, and transcriptional activation is observed upon its deletion. Conversely, mutation of this region to the subtype A/C 7-nt AP1 sequence enhances latency establishment [23].

The core promoter contains the essential elements for basal transcriptional activity, including a TATA box [69], an unconventional pyrimidine rich initiator region (Inr) [70], and three tandem GC-rich binding sites for Sp1 [71]. These Sp1 sites facilitate recruitment of the TBP subunit of TFIID to the TATA box, which is stabilized by the binding of TFIIA and TFIIB [72]. Together, these initiate the sequential assembly of the PIC, which will be discussed in more depth in Section 4.

The TAR element (+1 to +60) was discovered with the observed upregulation of reporter gene expression under the control of the 5’LTR in HIV-1-infected versus uninfected cells [27,57,73]. Sodroski et al. identified the precise location of this TAR element as well as the region of the HIV-1 genome, encoding Tat, required for transactivation. Later in vitro studies demonstrated that this TAR element forms a hairpin-like secondary structure to which Tat binds, to promote productive elongation of the HIV-1 genome [35,36,74,75].

Finally, sequences downstream of the TSS have also been shown to contain TF binding sites that modulate HIV-1 expression. These include 3x AP-1, NFATc2, C/EBP, AP-3, NFAT, DBF, and Sp1 binding sites (Figure 1). These factors modulate initiation and elongation directly. In addition, they also recruit chromatin remodeling factors to alter the accessibility of the DNA to the transcriptional machinery [22].

## 3. Epigenetic Modulation of HIV-1 Latency

Eukaryotic DNA is typically packaged into nucleosomes, which consist of 147 bp of DNA wrapped around an octamer that comprise pairs of four core histone proteins (H2A, H2B, H3, and H4) [76]. Nucleosome positioning is a dynamic process that closely associates with gene expression [77]. The accessibility of TFs to their promoter binding sites is affected by the position of nucleosomes, and nucleosome remodeling allows transcription machinery access to DNA. The positioning and stability of nucleosomes are regulated by three mechanisms.

First, chromatin remodeling complexes with ATPase domains actively move, deposit, or eject nucleosomes [78]. Second, histone N-terminal tails that protrude from the nucleosome core are subject to post-translational modifications (PTMs), such as acetylation, methylation, crotonylation, sumoylation, and ubiquitination [79,80]. These PTMs alter histone electrostatic charges, influencing histone-DNA and histone–histone interactions affecting nucleosome dynamics [81]. Additionally, the PTMs serve as a “histone code,” providing docking sites for proteins with cognate recognition domains that then affect gene expression. Third, DNA methylation on CpG islands also influences the chromatin environment. DNA methylation involves the transfer of a methyl group onto the C5 position of cytosines to form 5-methylcytosine. Methylation of CpG islands plays roles in epigenetic regulation of mammalian gene expression, through recruitment of repressor proteins or blocking the binding of positive TFs [82].

**Figure 2:**
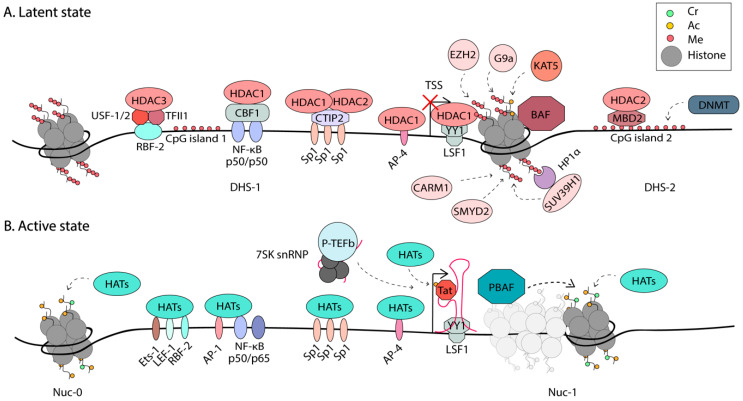
Epigenetic regulation of HIV-1 transcription. This is a simplified overview of some of the factors involved in the modulation of HIV-1 latency. (**A**) Several proteins have been implicated in either the establishment or maintenance of HIV-1 latency. These include TFs, such as RBF2, NF-κB p50 homodimers, CTIP2, AP-4, and YY1, which directly bind to DNA and serve as docking sites or recruitment factors for other proteins, such as HDACs and HMTs. HDACs remove acetyl groups from the histone tails in nucleosomes. HMTs add methyl groups to histone tails. HMTs involved in HIV-1 latency include the well-established EZH2, G9a, SUV39H1, and the newly associated CARM1 and SMYD2. KAT5 has been recently implicated in histone 4 acetylation at the promoter, associated with HIV-1 latency. The “histone code,” or combination of PTMs (e.g., acetylation and methylation) at the promoter, promotes latency by increasing the affinity of nucleosomes for DNA, reducing DNA accessibility and through recruitment of repressive factors. This includes the SWI/SNF chromatin remodeling BAF complex, which positions Nuc-1 just downstream of the TSS and blocks transcriptional elongation. DNA methyltransferases (DNMTs) are thought to be recruited to the promoter and hypermethylate of the two CpG islands, leading to recruitment of HDACs via an interaction of MBD2 with the methyl groups. Finally, P-TEFb, required for the transition from transcriptional initiation to elongation, is held inactive through its association with the 7SK snRNP. (**B**) During activation, transcriptional repressors are replaced with activators, including NF-κB p50/p65 heterodimers and HATs, such as p300/CBP and hGCN5. HATs can acetylate or crotonylate histones, promoting a more open chromatin and recruiting the SWI/SNF chromatin remodeling PBAF complex. PBAF repositions Nuc-1 further downstream of the TSS, to allow transcription elongation. Furthermore, Tat is acetylated by p300 and binds to the secondary structure of the nascent TAR RNA, produced by the initiation of competent RNAPII. Tat can extract active P-TEFb either directly from free 7SK snRNP, or from 7SK snRNP tethered to the promoter, via an association with KAP1.

All of these three mechanisms have been implicated in the regulation of HIV-1 expression. A summary of the epigenetic regulation of HIV-1 is shown in Figure 2.

### 3.1. Chromatin Remodeling

Certain DNA sequences are more amenable to nucleosome formation. It has been proposed that highly regulated promoters have evolved DNA sequences that are conducive to dynamic competition between nucleosome formation and proximally paused promoter RNAPII [83]. The HIV-1 promoter is a good example of this, as in silico predictions suggest that the DHS-1 region that contains multiple TF binding sites, has a higher propensity for histone binding than the region occupied by Nuc-1 [53]. However, this region is maintained nucleosome-free by the SWI/SNF chromatin remodeler Brg-associated factor (BAF) [53,54]. BAF helps position Nuc-1, in an ATP-dependent manner, to the more energetically unfavorable region, just downstream of the TSS, posing a block to transcriptional elongation [53]. Upon activation, HATs such as p300/CBP and hGCN5 are recruited to the LTR and acetylate both Tat and histones, loosening the DNA around nucleosomes [41,84,85]. It is well-accepted that Nuc-1 is remodeled during activation to allow elongation [44,45]. This function is proposed to be performed by the chromatin remodelers polybromo-associated BAF (PBAF) and the FACT complex (Facilitates Chromatin Transcription) [53,84]. BAF and PBAF share several subunits, including the Brg1 ATPase, but each complex has some unique subunits. The activating role of PBAF in HIV transcription was supported by studies on PBAF recruitment to the LTR by acetylated Tat [53,84]. In addition, depletion of the PBAF-specific subunit, BAF180, but not the BAF-specific BAF250, reduces Tat-mediated transactivation [53]. Furthermore, studies using the Tat inhibitor, dCA, showed depletion of PBAF-specific subunits at the LTR during dCA-mediated latency [86]. On cellular genes, the FACT complex is thought to act as a chaperone of H2A-H2B heterodimers during chromatin remodeling, to facilitate transcriptional elongation [87]. Components of the FACT complex were found to associate with the HIV-1 LTR and regulate transcription [84,88,89]. A full understanding of the players and mechanisms that promote Nuc-1 remodeling at the HIV-1 LTR to trigger transcriptional elongation is still outstanding. Some of these regulatory factors might prove to be suitable targets in block-and-lock approaches for a functional cure.

While BAF and PBAF share several subunits, they have opposite functions, and designs of specific inhibitors targeting their unique subunits might allow for specific modulation of latency. The Bromodomain-containing protein 4 (BRD4) has been shown to modulate BAF. The role of BRD4 in HIV-1 is complex, having both positive and negative regulatory effects on HIV-1 transcription [90,91,92,93,94,95,96]. BRD4 has two main isoforms [90], both containing Bromodomain-and-Extra-Terminal (BET) domains that mediate interactions with acetylated histones [97]. The long isoform, BRD4L, contains an extended C-terminal end that includes a P-TEFb interacting domain (PID). The short isoform, BRD4S, lacks the PID. In the absence of Tat it has been proposed that BRD4L delivers P-TEFb to latent proviruses, through the interaction of BRD4 with acetylated histones and the mediator complex, promoting productive elongation [98,99]; however, these studies were performed with HeLa cells and still need validation in a more relevant cell model of HIV latency. On the other hand, BRD4L has been shown to suppress HIV-1 transcription during Tat transactivation by competing with Tat for P-TEFb recruitment [95,96]. Recently, BRD4S was recently shown to also promote HIV-1 latency via its interaction and recruitment of BAF [90]. Inhibition of the BET domain of BRD4 by the small molecule JQ1 treatment disrupts the recruitment of the BAF complex, resulting in HIV-1 reactivation [92,100]. The BRD4S interacts directly with BRG1 in the BAF complex and this binding is abrogated by JQ1, resulting in marked Nuc-1 remodeling and HIV-1 reactivation. The activity of JQ1 was dependent on the presence of the BAF-specific BAF250 subunit, but not the PBAF-specific BAF180 subunit, suggesting JQ1 selectively inhibits the BAF complex over the PBAF complex. However, JQ1 inhibits other bromo-domain containing proteins, such as BRD2, and has off-target effects [97]. The use of JQ1 might be hampered by observed growth defects in cord-derived MSCs [101], as well as lymphoid and hematopoietic toxicity, memory deficits, and weight loss in mice [102,103]. More selective BAF inhibitors were recently identified, targeting the ARID1A (also known as BAF250) subunit [91]. These compounds demonstrated low toxicity and resulted in HIV-1 reactivation in both cell lines and primary cell models of latency, through reduced nucleosome occupancy at the 5’LTR. Additionally, these inhibitors enhanced the activity of several clinically approved LRAs [91]. These compounds support the possibility of selectively targeting the chromatin remodelers, BAF or PBAF, to modulate HIV-1 transcription.

### 3.2. Post-Translational Modifications of Histones

Different histone marks are associated with specific genomic domains, such as promoters, enhancers, and heterochromatin. This is no different for HIV-1 and has been extensively reviewed elsewhere [19,20,22]. The most well-characterized PTMs implicated in HIV-1 transcription are histone acetylation and methylation. Histone acetylation is catalyzed by a group of enzymes called HATs, whereas these acetyl groups are removed by histone deacetylases (HDACs). Histone methylation is attained by histone methyltransferases (HMTs). Histone acetylation is generally associated with active transcription, for instance, histone 3 lysine 27 acetylation (H3K27Ac) and H3K9Ac [104]. H3K27Ac, primarily mediated by the p300/CBP HAT [105,106,107], was shown to alter downstream transcription kinetics by as much as 50% in live single cell analysis [79]. H3K27Ac presence at promoters improved the recruitment of the TF, GR, and increased the levels of pSer2 RNAPII C-terminal domain (CTD), promoting the transition of RNAPII from initiation to elongation [79]. Of note, these studies were performed in mouse adenocarcinoma cells. Recently, components of the super elongation complex (SEC), which promote transcriptional elongation, were found to interact with H3K27Ac residues in pull-down assays, in HeLa cells [108]. Furthermore, higher levels of H3K27Ac at Nuc-1 in the 2D10 Jurkat cell line model of latency were observed upon activation [108]. Together, these studies support a positive role for H3K27Ac in HIV transcription. Methylation plays distinct roles in transcription regulation, depending on which histones and residues are modified. For example, active promoters are associated with histone 3 lysine 4 trimethylation (H3K4me3) and active transcription units with H3K79me3 and H3K36me3 [109]. On the other hand, methylation of H3K27, H3K9, and H4K20 are associated with transcriptional silencing [110]. Modulation of PTMs on the HIV-1 LTR has been explored to reactivate latent proviruses in shock-and-kill cure approaches, including the use of HDAC inhibitors Panobinostat, Vorinostat, and Romidepsin in clinical trials [111,112,113,114,115]. It is likely that a combination of approaches targeting multiple steps of HIV-1 transcription will be needed to synergistically modulate HIV-1 transcription in shock-and-kill or block-and-lock approaches for an HIV-1 cure. Here, we review some of the most recently discovered roles played by PTMs in HIV-1 latency.

The BRD4 BET consists of two bromodomains (BD1 and BD2) and one extra-terminal (ET) domain. BD1 and BD2 bind acetylated histones H3 (e.g., H3K4acK9ac) and H4 (e.g., tetra-acetylated H4 at K5, K8, K12, and K16) [116]. Lysine acetyltransferase 5 (KAT5) preferentially acetylates H3 Lys-14 and H4 Lys-5, -8, -12, and -16 of H4 in a cell-free system [117]. Recently, some light was shed on the “histone code” recognized by BRD4 and the writer, KAT5, that generates this code. ChIP against acetylated H3 and H4 revealed that latent HIV-1 LTRs contain low levels of AcH3 and high levels of AcH4, which correlate with the recruitment of BRD4 and the inhibition of Tat transactivation [118]. KAT5 was found to promote HIV-1 latency via its acetylation of H4 on the HIV-1 LTR. Suppression of KAT5 reduced AcH4, but not AcH3 marks, and inhibited BRD4 binding. KAT5 suppression was shown to increase SEC recruitment and reversed and delayed the establishment of latency in cell line models. These results were confirmed in primary cells from ART-treated patients [118]. BRD4 and KAT5 thus provide attractive targets for HIV-1 reactivation approaches, while promotion of AcH4 over AcH3 acetylation could be an avenue for viral silencing.

As mentioned above, H3K27Ac is a positive regulator of HIV-1 transcription. The Feng group performed a peptide pull-down/mass spectrometry (MS) analysis that identified the SEC associated with H3K27Ac [108]. H3K27Ac was shown to stimulate H3R26 methylation, which interestingly abrogated SEC binding. Inhibition of CARM1 (Coactivator-associated arginine methyltransferase 1), the enzyme that catalyzes H3R26me, with a small molecule inhibitor, results in HIV-1 reactivation in both cell line and primary CD4^+^T cell models of latency [108]. They established a working model where the SEC is recruited to H3K27Ac and further stimulates H3R26 methylation, subsequently abolishing SEC recruitment in a negative feedback loop. CARM1 inhibition was shown to act synergistically with other LRAs, possibly providing a novel target for HIV-1 eradication strategies [108].

Histone lysine side chains can be modified by the addition of one, two, or three methyl groups. This process is mediated by different HMTs, three of which have been associated with the HIV-1 LTR: EZH2, G9a, and SUV39H1. The HMT Enhancer of Zeste (EZH2), which is part of the Poylcomb repressive complex 2 (PRC2) catalyzes H3K27me3 and was shown to be involved in the maintenance of HIV-1 latency in Jurkat T cells [119]. Both PRC2 and euchromatic histone-lysine *N*-methyltransferase 2 (EHMT2) also known as G9a are highly enriched at the HIV-1 5’LTR in latently infected cells and are displaced upon reactivation [21]. G9a catalyzes dimethylation of H3K9 and SUV39H1 trimethylates this residue. The inhibition of HMTs has been shown to reactivate HIV-1 transcription and they also act synergistically with IL-15 and suberanilohydroxamic acid (SAHA) treatment, an HDAC inhibitor [21]. Due to their important role in latency, the Ott group performed an RNAi-based screen of all human HMTs to identify the novel players in HIV-1 regulation [120]. SMYD2 (SET and MYND domain-containing protein 2) was identified as a repressor of HIV-1 transcription. SMYD2 mediates H4K20me1 methylation at the HIV-1 LTR, a mark which has been shown to play roles in transcriptional repression [121]. H4K20me1 is recognized by the chromatin reader L3MBTL1, a protein with known roles in chromatin compaction [122]. Boehm et al. showed that L3MBTL1 is enriched on latent LTRs and is depleted upon reactivation [120]. Thus, it is proposed that the SMYD2-dependent recruitment of L3MBTL1, via H4K20me1, is involved in establishing and maintaining HIV-1 latency.

Histone crotonylation was recently shown to play an important role in the modulation of HIV-1 latency [123]. Lysine crotonylation (KCr) is a newly discovered histone PTM catalyzed by the HAT p300/CBP and is specifically enriched at active promoters. In fact, KCr has been shown to be a more potent activating mark than AcH [124]. Crotonyl-CoA serves as a substrate for histone crotonylation, thus the levels of intracellular Cr-CoA influence the levels of histone crotonylation and the transcriptional output of a cell [124]. The transcriptional upregulation upon enhanced crotonylation might be associated with the recent discovery of the high affinity interaction of the AF9 YEATS domain, with histone crotonylation [125]. AF9 is part of the SEC that is recruited to the stalled polymerases just downstream of the TSS, to allow productive elongation. Whether AF9 plays a role in SEC binding to crotonylated residues at the HIV-1 LTR remains to be determined.

HIV-1 reactivation was observed when the Cr-CoA producing enzyme, acyl-CoA synthetase short-chain family member 2 (ACSS2) was overexpressed in the cell models of latency [123]. HIV-1 reactivation was associated with a reprogramming of the local LTR chromatin environment, with increased KCr and AcH, and reduced histone methylation. Conversely, suppression of ACSS2 reduced HIV-1 reactivation. Notably, acute SIV infection is associated with an upregulation of ACSS2 in vivo in the gut of rhesus macaques, which decreased the chronic stage of infection [123]. Importantly, ACSS2 is upregulated upon treatment interruption in ART-suppressed individuals [126]. ACSS2 is an essential enzyme in controlling lipid or fatty acid metabolism [127] and has been shown to play important roles in T cell function [128]. HIV-1 expression has been shown to alter lipid metabolism in CD4^+^ T cells [129]. Altogether, these findings suggest that fatty acid metabolism can modulate HIV-1 transcription, through the induction of ACSS2. Thus, ACSS2 could serve as a target for modulating HIV-1 expression, either to promote transcriptional silencing or reactivation. Investigation of the role of ACSS2 and crotonylation in HIV-1 transcription deserves further attention.

### 3.3. DNA Methylation of the HIV-1 Genome

Two CpG islands flank the HIV-1 TSS and have been shown to be hypermethylated in latently infected cell lines and primary CD4+T cells [130,131]. Induction of viral gene expression with LRAs results in demethylation of these islands. Methyl-CpG binding domain protein 2 (MBD2) was identified in a cDNA library screen to play a role in latency, through its recruitment of HDAC2 to the CpG island, downstream of the TSS [131]. Methylation was shown to accumulate, once initial silencing has already been achieved [132]. Latency in the absence of DNA hypermethylation on the HIV-1 LTR is leaky, suggesting it acts as an additional mechanism of silencing. Furthermore, analysis of DNA methylation in primary cells from long-term non-progressors who were not on ART revealed high levels of intragenic hypermethylation of HIV-1 DNA, with conversely low levels of promoter methylation, showing the importance of analyzing the complete HIV-1 genome in latency studies [133].

## 4. Early Stages of Transcription

**Figure 3:**
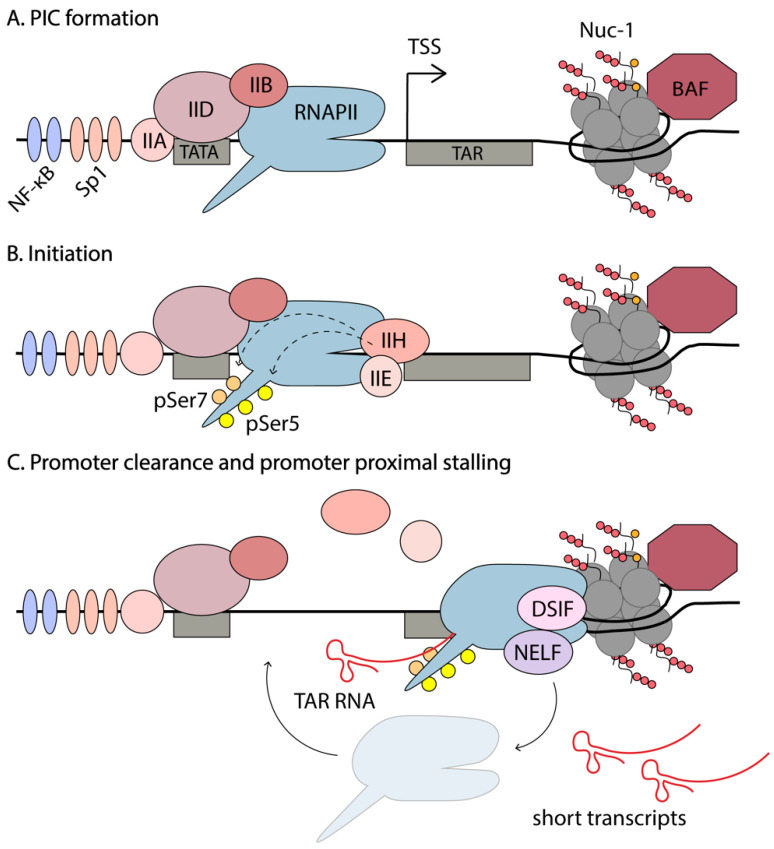
Pre-initiation complex formation and promoter proximal stalling. Even under latent conditions, there is an accumulation of RNAPII at the TSS. (**A**) The binding of host TFs, such as NF-κB and Sp1, and general transcription factors TFIIA, TFIID, and TFIIB to their cognate sites, form the PIC on the HIV-1 promoter. Hypophosphorylated RNAPII is recruited to the PIC through interactions with these TFs. In this state, RNAPII is unable to initiate transcription. (**B**) The final general transcription factor to be recruited to the PIC is TFIIH. TFIIH binds directly to the DNA and the XPB subunit creates negative supercoiling and serves as a molecular wrench, threading the DNA through the RNAPII active site. The CDK7 subunit of TFIIH phosphorylates Ser7 and Ser5 of the RNAPII C-terminal domain (CTD) to activate the polymerase to begin transcription. (**C**) Once activated, RNAPII clears the promoter, transcribing the TAR RNA, but pausing just after due to the occlusion by Nuc-1 and the presence of the negative regulators DSIF and NELF. The paused polymerase then dissociates from the DNA and releases the short RNA transcript. RNAPII is then free to participate in PIC formation once again. Thus, there is an accumulation of RNAPII at the promoter of latent proviruses due to both occluded RNAPII in the PIC and stalled polymerases just after the TSS.

In a recently published study, Yukl et al. utilized droplet digital PCR (RT-ddPCR) technology to assess the abundance of different HIV-1 transcripts in primary CD4^+^ T cells from HIV-1-infected individuals [43]. This study reported higher levels of short TAR transcripts than previously observed, with less sensitive RT-qPCRs. This method was used to investigate blocks of transcription in these cells stimulated by TCR activation or ex vivo LRA exposure, to induce HIV-1 expression. They found that the main block of HIV-1 transcription in latently infected cells is not initiation, but rather a series of blocks of elongation, completion (which refers to distal transcription and polyadenylation), and multiple splicing. An average of 20 copies of TAR RNA per copy of integrated proviral DNA was observed. In the absence of Tat, efficient initiation by RNAPII is observed, but it rapidly stalls after transcribing the TAR RNA. The polymerase is believed to disassociate and initiate novel rounds of transcription. Since the secondary structure of TAR limits its degradation, there is accumulation of these short abortive transcripts. ChIP-seq data shows enrichment of RNAPII at the HIV-1 promoter, just downstream of the TSS [47]. Together these results suggest that, in the absence of T cell activation, when host TFs such as NF-κB and NFAT are sequestered in the cytoplasm and unable to participate in the upregulation of gene expression, there is still recruitment of the PIC and some degree of promoter escape to transcribe the first ~100bp of the HIV-1 genome, including the TAR RNA. Due to a scarcity of TFs and Tat, RNAPII stalls and accumulates at Nuc-1, destabilizes, and disassociates, creating short abortive transcripts. The events involved in transcription initiation are summarized in Figure 3.

As mentioned in Section 2, the HIV enhancer and core promoter are comprised of two NF-κB binding sites, three tandem Sp1 sites, a TATA box and an initiator element. PIC assembly on the LTR starts with the synchronized binding of the general transcription factors, namely the TATA-binding protein (TBP) to the TATA box (part of TFIID), TFIIA, TFIIB, TFIIF, and the mediator complex [29]. PIC assembly is also enhanced by activator TFs such as NF-κB and Sp1 binding to the LTR. PIC assembly occurs independently of Tat; however, once Tat is made, it is enhanced [29,39]. For example, Tat recruits TBP to the HIV-1 LTR in the absence of other TFIID components, such as TAFs, through an interaction with P-TEFb [39]. PIC assembly is completed by recruitment of RNAPII and the final general transcription factors, TFIIE and TFIIH [134,135].

The role of the mediator complex in HIV-1 transcription is still not clear. It plays key roles in metazoan transcription and is composed of ~30 protein subunits that are organized into head, middle, tail, and kinase modules [136]. Certain components of the mediator have been shown to positively regulate HIV-1 transcription and are found in the PIC on the HIV-1 LTR [39,137]. For instance, Tat has been shown to interact directly with the Med14 component of mediator, and the knockdown of Med14 inhibits Tat-mediated transactivation of HIV-1 [137]. On the other hand, the kinase module (composed of two subunits Med12 and Med13 and the kinase CDK8, or its paralog CDK19) are thought to be dispensable for HIV-1 transcription [136]. This is evidenced by small molecule inhibition or knockdown of these proteins in cell line models of HIV-1 latency [138,139]. Additional research would benefit our understanding of the role of mediator in HIV-1 PIC formation, initiation and elongation, particularly in biologically relevant systems such as primary CD4^+^T cells.

RNAPII is recruited to the PIC hypophosphorylated, and phosphorylation by TFIIH provides RNAPII activation. TFIIH consists of ten subunits comprising the core module (XPB, XPD, p8, p52, p34, p44, and p62) and the CDK-activating kinase (CAK) module, consisting of MAT1 and CDK7 with its partner Cyclin H [135,140,141]. Recruitment of TFIIH to the PIC has been proposed as the rate-limiting step in the reactivation of latent HIV [61]. TFIIH has been shown to participate in promoter clearance and to be released from the transcriptional complex before the TAR RNA is synthesized [40]. TFIIH plays two important roles in initiation—(1) creates negative supercoiling in the DNA to facilitate promoter opening to lock the DNA substrate into the active cleft of RNAPII and (2) creates a competent transcribing complex that can initiate transcription and escape the promoter, through phosphorylation of the CTD of RNAPII [135,142]. The largest subunit of RNAPII, Rpb1, contains 52 repeats of the heptapeptide-sequence Y_1_S_2_P_3_T_4_S_5_P_6_S_7_ in its CTD tail. This repetitive sequence serves as a binding scaffold for factors involved in transcription and serves as a hotspot for post-translational modifications that regulate transcription [143]. Phosphorylation of the three serine residues is associated with different phases of transcription. RNAPII recruited in PICs is unphosphorylated, and phosphorylation of Ser7 by CDK7 primes the polymerase for further phosphorylation of Ser5 and initiation of mRNA synthesis [143]. pSer7 levels remain high throughout transcription, whereas pSer5 are high at the TSS but decrease rapidly towards the end of the gene [143]. pSer2 are associated with the transition from initiation to elongation and levels increase towards the end of genes, consistent with the requirement of pSer2 for recruitment of 3’RNA processing factors [144].

There are five cyclin-dependent kinases (CDKs) that play major roles in the regulation of eukaryotic transcription—CDK7/Cyclin H as part of the TFIIH general transcription factor complex; CDK8/Cyclin C as part of the mediator complex; CDK9/Cyclin T1/T2 as part of the active form of P-TEFb; CDK12/Cyclin K, and CDK13/Cyclin K [145]. Promiscuity exists in these kinase specificities; however, it is generally accepted that CDK7 initiates transcription through Ser7 and Ser5 phosphorylation of the RNAPII CTD. CDK7 also activates other CDKs through phosphorylation of their T-loops [95,146]. CDK9 functions as an early elongation kinase to overcome promoter proximal pausing, through phosphorylation of Ser2 on RNAPII CTD, as well as two elongation factors associated with paused RNAPII (NELF and DSIF) [147,148,149]. CDK8 generally plays a suppressive role as part of the mediator complex, though it is known to activate transcription of certain cellular genes [150]. Importantly, it was recently demonstrated that knockdown of CDK8 (and its paralog CDK19) had no effect on HIV-1 transcription [138,139]. CDK12/CDK13 has been shown to be involved in Ser2 phosphorylation towards the midpoint and 3’ ends of genes, and play a role in RNA processing and splicing [143,151,152]. CDK13 was also shown to interact with Tat and increase multiply spliced viral mRNAs [153]. The role of CDK12/CDK13 in HIV-1 transcription is yet to be established. For the purposes of this review, we are focusing on the role of CDK7 and CDK9 and their interacting partners in HIV-1 transcriptional regulation, and how these might be modulated in cure approaches.

In summary, upon cytokine stimulation or TCR activation, TFs, such as NF-κB and NFAT, translocate into the nucleus and bind to their consensus sequences. This results in the recruitment of additional RNAPII and general TFs, to increase transcriptional initiation and promoter escape [61]. Stalling of RNAPII at Nuc-1 is relieved by inefficient recruitment of P-TEFb by host factors, such as NF-κB and BRD4 [98,154]. There is also evidence that BRD4 might serve as an atypical kinase that binds to RNAPII and phosphorylates Ser2 residues [155]. However, it is not clear whether or not this results in elongation or if BRD4 is capable of phosphorylating DSIF and NELF; additional steps required for elongation. Ultimately, elongation leads to synthesis of full-length HIV-1 mRNAs, which are spliced to produce Tat. Once Tat is made, there is robust stimulation of RNAPII elongation and efficient viral production. Recently, the D’Orso group proposed a novel HIV-1 transcription circuit that is regulated at three different levels [156]. First, in resting cells, during homeostasis, latent provirus have low levels of non-productive RNA synthesis that result in short, abortive transcripts, and this is termed the “basal” state. At this stage Tat is not yet produced and thus there is no transactivation of the virus. In the “host” phase, cells are exposed to immune stimulation and TFs such as NF-κB and NFAT, “boosting” transcription and resulting in some Tat production. It is proposed that this phase poses “transcriptional circuit fragility” because if a certain threshold of Tat is not yet produced, the system is turned back off and the virus remains silent. If sufficient Tat is produced from the “boost” it can set up the positive feedback loop that leads to robust viral production, establishing the “viral” phase of transcription sustained by Tat [156].

## 5. Elongation of Transcription

A general feature of metazoan transcription is promoter-proximal pausing of RNAPII. Only a small proportion of initiation events results in full length, mature mRNAs. After efficient recruitment of the PIC and initiation of transcription, the RNAPII complex pauses shortly after the TSS and remains poised for elongation. This step is tightly regulated by several negative and positive elongation factors that are discussed below. The position of Nuc-1 is maintained in an unfavorable position by the chromatin remodeling complex, BAF, and paused RNAPII is suggested to play a role in this, physically occluding Nuc-1 [53,83]. This maintains chromatin in a permissive state, allowing access of TFs to the DNA. The promoter-proximal pausing of RNAPII also permits rapid changes in gene expression, in response to internal and external stimuli, circumventing the steps required for PIC assembly [157,158,159]. Finally, promoter-proximal pausing functions as a checkpoint for coupling elongation with RNA processing events such as 5’ capping, splicing, and polyadenylation to produce mature mRNA [83].

Promoter-proximal stalling is especially evident on the HIV-1 5’LTR. The presence of multiple TF binding sites results in successful PIC recruitment, even in latently infected cells, as evidenced by an accumulation of RNAPII at the TSS by ChIP-seq [47]. However, the elongation phase is blocked due to the presence of negative elongation factors and the suppressive Nuc-1, and an absence of positive elongation factors, including Tat. The position where RNAPII usually stalls along genes is well conserved across the genome, throughout the species [47].

Major pause sites for eukaryotic genes include the +20 to +60 nt region from the TSS, as well as at −90 to −110 nt region corresponding to divergent transcription, where two polymerases initiate transcription at the TSS in opposite directions and pause at the +1 and −1 nucleosomes [160]. The RNAPII pause sites on the HIV-1 genome were extensively mapped by the Karn group [47]. They found several sites that are shared with cellular genes and some unique to HIV-1. An RNAPII peak was observed at +11 nt from the TSS, which likely corresponded to occluded PIC in latent proviruses that require TFIIH to initiate transcription [61]. The major pause sites seen in cellular genes at +50, +220, and +330 nt are also observed on the LTR. However, an extra pause site was present directly downstream of TAR at +120 nt [47]. This provides additional control to HIV-1 gene expression. In fact, in the absence of Tat, elongation from the HIV-1 promoter is more restricted than in cellular genes, as only ~10% of initiated RNAPII elongates, as compared to 20–30% for paused cellular genes [161]. In the presence of Tat; however, the efficiency at which RNAPII elongates to transcribe the HIV-1 genome increases to approximately 75%.

Due to the premature stalling of the early elongation complex, short transcripts are formed. On the HIV-1 LTR, the nascent RNA forms a functionally important, highly stable secondary structure, TAR, from the first 59 nt. Although cellular genes also display promoter-proximal pausing, there is no accumulation of these short abortive transcripts, as they are rapidly degraded. Another unique feature of HIV-1 transcription, revealed by ChIP-Seq experiments, is that HIV-1 has 3–10 fold lower levels of negative strand synthesis (divergent transcription) from the 5’LTR compared to that observed on cellular genes [47]. This suggests that HIV-1 has an unusual bias towards positive strand synthesis, though the reason for this remains unclear.

### 5.1. P-TEFb Regulation

Promoter-proximal pausing is overcome through the action of the positive elongation factor P-TEFb, which consists of a heterodimer of the kinase CDK9 and its associated Cyclin T1 or Cyclin T2 (CycT) [162]. As mentioned in Section 4 above, this is achieved through the phosphorylation of two RNAPII-associated elongation factors, DSIF and NELF. ChIP-seq experiments revealed that DSIF and NELF share almost overlapping peaks with RNAPII near the TSS [163]. DSIF consists of two subunits, Spt4 and Spt5, and causes promoter-proximal pausing of RNAPII [164]. However, upon phosphorylation of the Spt5 subunit by the CDK9 subunit of P-TEFb, DSIF becomes a positive elongation factor that tracks along with RNAPII, through the gene body [148,163]. A peak of NELF is observed at the TSS along with the paused RNAPII, but is not detected in the gene body [47,163]. NELF consists of four subunits, NELF-A, B, C, and E, which together bind to RNAPII and stall the polymerase [147,165]. Phosphorylation of the NELF-A and NELF-E subunits by P-TEFb results in their disassociation from the transcription complex to permit RNAPII elongation [166,167].

These two phosphorylation events triggered by P-TEFb are essential for proper gene expression, thus the need for tight control of P-TEFb. P-TEFb can be modulated through transcriptional and translational control, post-translational modifications, miRNAs, and modulation of protein turnover. For example, in resting CD4^+^T cells, the levels of active CDK9 are low-to-undetectable, accumulating in an inactive complex with cytoplasmic Hsp90/Cdc37 chaperones [168,169]. This complex keeps CDK9 stable in the cytoplasm, prior to CycT1 upregulation by T cell activation. CycT1 protein levels are downregulated by microRNAs and this is reversed upon T cell activation [170]. Recently, the highest levels of CycT1 were measured in maximally activated uninfected memory CD4^+^T cells, but the CycT1 levels dropped upon HIV-1 replication [168]. Downregulation of P-TEFb function and levels of CycT1 precede the return of HIV-1-infected CD4^+^T cells’ return to quiescence. This in turn leads to suppression of HIV-1 transcription and contributes to the establishment of latency.

**Figure 4:**
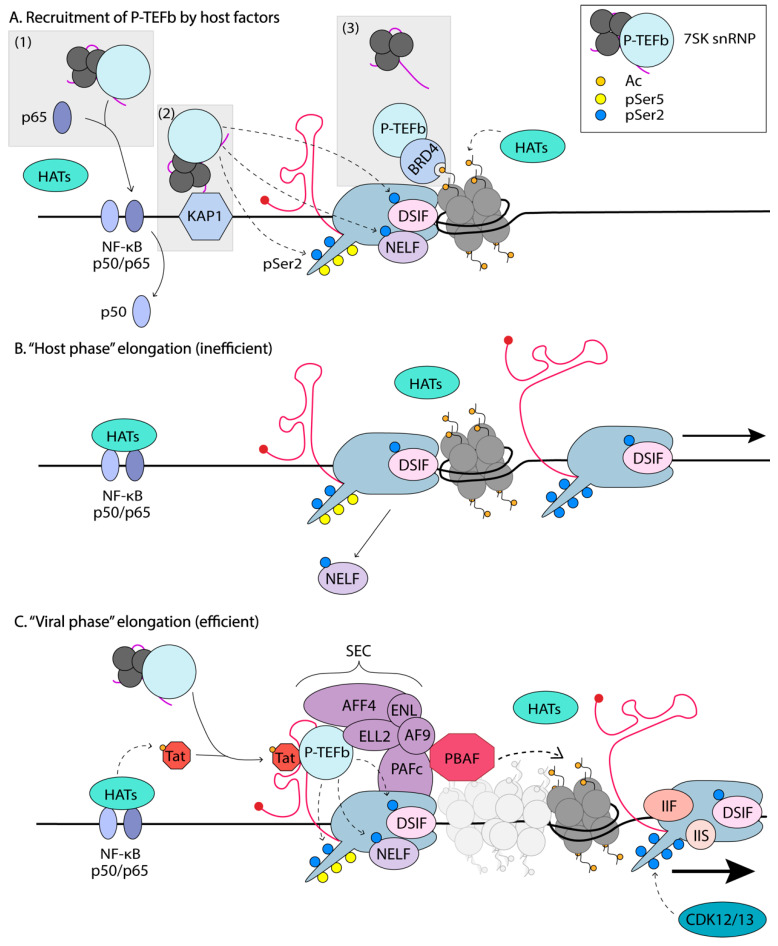
Regulation of transcription elongation. (**A**) In activated cells, in the absence of Tat, TFs such as the NF-κB p65 subunit translocate to the nucleus and bind to their consensus sequence. In this process they displace transcriptional repressors, e.g. p50 homodimers and associated HDACs. P-TEFb is recruited (shown by the grey boxes) to the paused RNAPII at promoters, through either a direct interaction with p65 (**1**), indirectly through recruitment of the 7SK snRNP to promoter DNA by KAP1 (**2**) or through extraction from the 7SK snRNP by BRD4, which binds to acetylated histones (**3**). Once in the proximity of the paused RNAPII, the CDK9 subunit of P-TEFb phosphorylates Ser2 residues on the RNAPII CTD, as well as NELF (releasing it from RNAPII) and DSIF (converting it to an elongation factor). (**B**) In the absence of Tat, the recruitment of P-TEFb is inefficient and results in limited transcription elongation (due to the repressive Nuc-1 positioning), but ultimately leads to a production of some full length HIV-1 mRNAs, which are spliced to produce all transcripts for the proteins that are necessary for the viral lifecycle, including Tat. The level of full-length mRNA production in this “host phase” is dependent on the availability of host TFs, such as NF-κB and NFAT. The efficiency of the “host phase” determines the amount of Tat produced, and whether a certain threshold will be met to enter the “viral phase”. (**C**) Tat is acetylated by the HAT p300, which enhances the interaction of the CycT1 subunit of P-TEFb with the Tat-TAR recognition motif. Phosphorylation of the CTD, DSIF, and NELF by P-TEFb, triggers the transition of promoter-proximal paused RNAPII to productive elongation. Acetylated Tat also recruits the SEC and the chromatin remodeler PBAF, which further promotes productive elongation through repositioning of Nuc-1. Efficient elongation is maintained through interactions with TFIIS and TFIIF, which relieve polymerase stalling in the gene body. The CTD Ser2 of RNAPII is further phosphorylated, likely by CDK12/13, to promote interactions with RNA splicing and processing factors, leading to efficient gene expression.

P-TEFb is also regulated by a unique mechanism involving the reversible association with the 7SK small nuclear ribonucleoprotein (snRNP) (Figure 4). In rapidly growing cells, such as HeLa cells, ~90% of P-TEFb is found in an inactive complex with the 7SK snRNP [171]. The 7SK small nuclear RNA, folds into a 3D scaffold that is stabilized by the La-related protein 7 (LARP7), the 7SK snRNA methylphosphate capping enzyme (MePCE), and a homodimer of the kinase inhibitor hexamethylene bisacetamide-inducible protein 1/2 (HEXIM). This complex binds to two P-TEFb molecules. The 7SK snRNP provides an exchangeable pool of activated P-TEFb that can be readily utilized to relieve promoter-proximal pausing and increase gene expression. However, in resting CD4^+^T cells, the levels of the 7SK snRNP are low [172], and thus not likely the main mechanism of P-TEFb control, instead P-TEFb protein levels are restricted, through miRNA-mediated downregulation of CycT1 and sequestration of CDK9 by Hsp90/Cdc37. Upon prostratin stimulation of resting CD4^+^T cells, the 7SK snRNP and P-TEFb levels increase [172], at which point the 7SK snRNP might then function to regulate P-TEFb levels.

Phosphorylation of Thr-186 CDK9 activation loop (T-loop) signals dissociation of CDK9 from the Hsp90/Cdc37 chaperones and facilitates assembly with CycT1, via intramolecular hydrogen bonding between pT186 and the CycT1 arginine triad and Glu-96, to form active P-TEFb [95]. In vitro kinase assays showed autophosphorylation of CDK9 T186 [173]; however, in cells, CDK7 has been implicated in activating pT186 [174]. Activated P-TEFb translocates to the nucleus and is incorporated into the 7SK snRNP, where CDK9 T-loop T186 phosphorylation is also a pre-requisite for assembly with HEXIM [175]. The inhibitory domain of HEXIM, which is exposed on binding to the 7SK snRNA, binds to the activated CDK9, preventing its inappropriate participation in transcription. The 7SK snRNP complex is free to diffuse in the nucleus and is thus able to deliver active P-TEFb to the sites of active transcription.

In order to participate in transcriptional elongation, P-TEFb must detach from the 7SK snRNP/HEXIM complex (Figure 4). In uninfected cells, this function is performed by BRD4, which is usually found to be associated with acetylated histone tails on active chromatin, thus delivering P-TEFb to the transcription site [176]. Acetylation of CycT1 by the HAT p300 in vitro has been shown to lead to the dissociation of P-TEFb from the 7SK snRNP [177]. Tat is also capable of extracting P-TEFb from the 7SK snRNP, by competing with HEXIM1 for binding to P-TEFb. Additionally, Tat has been shown to recruit the ubiquitin ligase, UBE20, to 7SK snRNP pools, to promote the non-degradative ubiquitination of HEXIM1 [178]. This results in the release of HEXIM1 from the complex and its sequestration into the cytoplasm, leaving P-TEFb free to participate in transcription elongation. The exchange of P-TEFb between the 7SK snRNP and Tat/BRD4 is also thought to be regulated by phosphorylation of T-loop Ser-175 of CDK9, and by the CDK7 kinase of TFIIH [95]. BRD4 was shown to interact with Ser-175; however, phosphorylation of this residue favors Tat’s interaction with P-TEFb over BDR4 [95].

### 5.2. Delivery of P-TEFb to Promoter-Proximal Paused RNA Polymerase II

P-TEFb can be delivered to paused RNAPII on the HIV-1 genome through several means (Figure 4). First, P-TEFb might be tethered to transcriptional activators that bind directly to the HIV-1 promoter, such as NF-κB via an interaction with the RelA subunit [154]. This mechanism was demonstrated during IL-8 gene activation, and thus, could in theory be an alternative mechanism of Tat-independent delivery of P-TEFb to the HIV-1 promoter. CDK9 is also recruited by c-Myc to its target cellular genes to activate their expression [163]. Second, P-TEFb can be recruited to promoters and enhancers through interaction with chromatin binding proteins such as BRD4 [94,98]. Third, HIV-1 directly recruits P-TEFb to its promoter by binding the CycT1 subunit to Tat and its high affinity interaction with the newly transcribed TAR RNA [179].

In addition to recruiting P-TEFb, Tat also associates with other SEC elongation factors. The SEC consists of transcription elongation activators/coactivators, including P-TEFb, ELL1/2, AFF4/1, ENL, and AF9, and different SEC subunit combinations make up distinct SEC complexes [180]. Tat was shown to increase the half-life of ELL2, thus stabilizing the SEC [171]. The SEC subunits are tethered to the RNAPII complex via the polymerase-associated factor complex (PAFc) [181]. PAFc also serves as a mediator to facilitate binding of other elongation factors to RNAPII, including DSIF and TFIIF. It is unclear how the SEC is recruited to the paused RNAPII in healthy, uninfected cells, but interactions of PAFc and AF9/ENL with chromatin have been suggested [181]. The MED26 subunit of the mediator complex also seems to play this role, interacting first with TFIID and then with the components of the SEC [182]. Recruitment of the SEC to genes in healthy cells might be tissue- or context-dependent and further work is needed to understand these mechanisms in detail.

Recently, through affinity purification followed by MS, the D’Orso group identified the Kruppel-associated box (KRAB)-interacting protein 1 (KAP1), as an interactor of the 7SK snRNP [183]. Through biochemical and genomic approaches, they showed that the KAP1-7SK snRNP complex is recruited to the majority of proximally paused promoter RNAPII and facilitates transcriptional pause release. HIV-1 is thought to exploit this complex for the transcription of its genome via the delivery of P-TEFb to NF-κB (Figure 4) [183]. The same research group proposed that HIV-1 has evolved a minimal transcriptional circuitry that bypasses host regulatory mechanisms for robust viral expression. However, this circuitry is fragile and dependent on the “host phase”, which primes the virus for activation [156]. In this model, HIV-1 is efficiently activated in response to immune stimulation, for example, through the concerted action of host TFs that boost PIC formation and initiation of transcription. During this host phase, TFs such as NF-κB translocate into the nucleus and bind to their consensus sequence on the HIV-1 promoter. Since NF-κB has been shown to bind P-TEFb and deliver it to paused polymerases [154], KAP1 could serve as an intermediate by delivering P-TEFb to the NF-κB at the promoter. This would allow the Tat-independent synthesis of full-length transcripts, and ultimately, the production of Tat. This would initiate the “viral phase,” where Tat drives productive elongation.

The transcriptional circuit is thus dependent on (1) the NF-κB associating with the promoter, (2) a KAP1 molecule binding near the transcriptional complex with an associated 7SK snRNP, and (3) delivery of P-TEFb from KAP1 to RNAPII via NF-κB, to phosphorylate Ser2 of RNAPII CTD, NELF, and DSIF, to elongate viral transcripts and produce Tat, which then bypasses steps 1 and 2 to establish a positive feedback loop [156]. This model was verified experimentally in primary and cell line models. The efficiency of the “host phase” was shown to determine the magnitude of the “viral phase,” and this could explain the large differences observed in the latency reversal observed in both primary models and ex vivo patient samples [184]. In contrast, previous work by Weinberger et al. used mathematical models to demonstrate that stochastic fluctuations in the level of Tat determined the proviral fate [45]. This was later shown to be independent of the cell state, where viral production was shown to persist, even as activated T cells return to quiescence [46]. D’Orso argues that their updated model integrates the effects of host cell factors and immune cell stimulation on the positive feedback loop established by Tat (basal-host-viral phases) rather than just the basal and viral phases. This was done by investigating how oscillations of KAP1 levels affect the heterogeneity of viral expression. Whether latency is ultimately dependent or independent on cell state remains to be fully established.

Regardless of how P-TEFb is recruited to the paused RNAPII, when all necessary elongation factors are present, RNAPII elongates HIV transcription proceeds at a steady rate of ~1.9kb/min [185], slightly lower than that of cellular genes with a rate of ~3.8 kb/min per two million base pairs [186]. Once promoter-proximal pausing escape is achieved, P-TEFb is no longer required for elongation and is rapidly sequestered back into the 7SK snRNP inactive complex, blocking undesired gene expression. For this, HEXIM1 associates with the 7SK RNA, allowing P-TEFb binding and inactivation [22]. Elongating RNAPII has the tendency to stall along the template DNA. Pausing is defined as a temporary state that is overcome with time, whilst RNAPII arrest cannot. Arrest of RNAPII is characterized by backtracking of the polymerase, where it moves backward on the DNA template, displacing the 3’ end of the RNA from its active site. Arrested polymerases eventually become subject to ubiquitination and degradation [171]. Factors involved in release of paused or arrested RNAPII are TFIIS and TFIIF. TFIIS rescues RNAPII from the arrested state by promoting cleavage of the nascent RNA to allow RNAPII to generate a new 3’ end of the RNA, correctly aligned to the template and the catalytic site [187]. TFIIF, in addition to its role in initiation, can also bind paused RNAPII and convert it back to the elongation-competent form [188] (Figure 4).

In summary, HIV-1 transcription, supported by cellular activation before Tat is produced, is triggered by intracellular signaling leading to translocation of TFs to the nucleus, chromatin modifications, and PIC assembly. Once activated by TFIIH, RNAPII initiates transcription and escapes the promoter, but rapidly stalls after transcribing the TAR RNA. Some P-TEFb is then recruited to the LTR by cellular factors, such as NF-κB, BRD4, or the KAP1-7SK snRNP complex already present at the promoter. P-TEFb phosphorylates RNAPII to promote synthesis of a few mature HIV-1 mRNAs [47]. This results in enough Tat production to recruit P-TEFb and SEC to RNAPII stalled at the TAR RNA [156], thus driving the positive feed-forward loop that results in robust HIV-1 expression,

## 6. Events Coupled to Elongation

**Figure 5:**
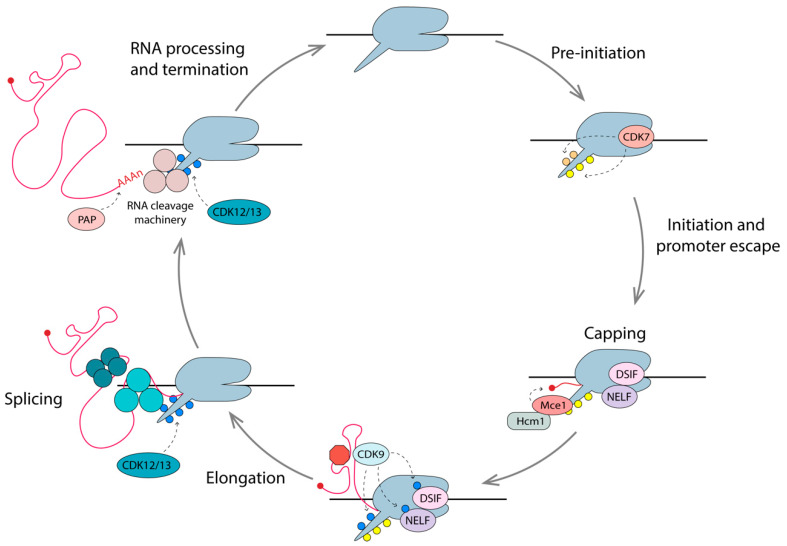
Transcription coupled events. Once the PIC has formed, and Ser7 and Ser5 phosphorylation of the RNAPII CTD by CDK7 (as part of TFIIH) allows initiation and promoter escape to occur. Shortly after transcription of the first 25–30 nt the 5’end of the nascent RNA is capped by the capping enzymes Mce1 and Hcm1. This process occurs more efficiently when RNAPII CTD is phosphorylated at Ser5 residues. Elongation is induced by phosphorylation events by CDK9, including CTD Ser2 phosphorylation. pSer2 is increased and RNAPII moves down the gene body by CDK12/13. This enhances recruitment of splice factors and production of fully spliced mRNAs. Finally, the recruitment of RNA cleavage factors are dependent on pSer2. The generation of cleaved 3’ends occurs 10–35 nt downstream of the polyA consensus sequence and the enzyme PAP adds 200–300 A residues to 3’OH group. Once the mRNA is released from RNAPII and CTD phosphorylation is removed by phosphatases, it is free to participate in the formation of the PIC to initiate new rounds of transcription.

Gene expression is proofed by coupling mRNA surveillance, export, processing, and maturation to transcription. The CTD tail plays a key role in coupling these events, as it is located at the pre-mRNA exit channel of RNAPII. The 52 heptad repeats, Y_1_S_2_P_3_T_4_S_5_P_6_S_7_, serve as docking sites for various factors and enzymes with key roles in these processes, and the post-translational modifications of the CTD also provide a vast combination of possibilities for modulation [171]. The combination of these modifications, known as the CTD code, coordinates transcription with pre-mRNA processing events through association or dissociation with various protein complexes, which are outlined below (Figure 5) [189].

### 6.1. Capping

The addition of a 7-methylguanylate (m^7^G) cap to the 5’ end of nascent pre-mRNA (including HIV-1 mRNAs), occurs once transcripts reach 25–30 nt in length [190]. The capping apparatus consists of a bifunctional triphosphatase-guanyltransferase (Mce1) and a methyltransferase (Hcm1), which physically interact with the Spt5 subunit of the DSIF in complex with phosphorylated RNAPII [191,192]. The presence of phosphorylated Ser5 on the RNAPII CTD stimulates the capping enzymes by 4-fold [190]. HIV-1 pre-mRNAs are efficiently capped when RNAPII experiences promoter-proximal pausing but are far less efficiently capped when RNAPII is unimpeded. Since NELF specifically recognizes RNAPII with pSer5 CTD and DSIF, it is thought that promoter-proximal pausing provides a window of opportunity for the recruitment and action of capping enzymes. Tat has been shown to bind Mce1 directly, enhancing co-transcriptional capping [193]. Once the transcript has been capped, and the elongation phase has begun, the levels of pSer2 on the CTD increases, due to phosphorylation by CDK12/13 [143,194,195].

### 6.2. Splicing

HIV-1 pre-mRNA can be spliced in several different ways to allow the expression of multiple proteins from a single transcript (Figure 1). In fact, over 50% of full length transcripts are spliced into >50 relevant mRNAs to produce the 10 HIV-1 proteins, using four donor sites and 10 acceptor sites [196]. Hyperphosphorylated RNAPII is also involved in the crosstalk between transcription and splicing. The association between pSer2 CTD and splice factors, including splicing regulatory (SR) proteins, Spt6 and U1-snRNP, ensures the stepwise assembly of the spliceosome close to the nascent pre-mRNA [197,198]. CDK12 (and homolog CDK13) has been shown to be the primary pSer2 kinase during the elongation phase of mammalian transcription, and is thus likely to play a role in HIV transcription [151]. Acetylated Tat is known to enhance the production of multiply spliced RNAs [199], and, interestingly, Tat was shown to interact with CDK13 to promote HIV-1 splicing [153]. Depletion of CDK12/13 results in a reduced expression of long cellular genes with multiple exons [200], as well as defects in RNA processing [152]. Flag-tagged CDK12 and CDK13 have also been shown to interact directly with various splicing factors [152]. Collectively, these studies support the idea that transcription and splicing are coupled, and this is mediated through interactions of the spliceosome components with pSer2 CTD of RNAPII on the nascent RNA.

### 6.3. Pre-mRNA Cleavage and Polyadenylation

As with capping and splicing, cleavage and polyadenylation of pre-mRNAs are generally coupled with transcription. In fact, recruitment of the cleavage stimulation factor 64 (CstF-64) is dependent on pSer2 RNAPII CTD [201]. Cleavage of the 3’ end of pre-mRNA occurs 10–35 nt downstream of the consensus polyA signal (AAUAAA), where 200–300 adenosines are added to the newly generated 3’-OH terminal end, by the enzyme poly(A) polymerase (PAP) [202,203].

In summary, efficient synthesis of the mature mRNA is facilitated by the interactions of the RNAPII CTD with various RNA processing factors, such that nascent RNAs are capped, spliced, cleaved, and polyadenylated as the transcription machinery moves along the gene body.

## 7. Therapeutic Strategies and Targets, Recent Advances

Historically, HIV-1 cure research has focused on the total elimination of the latent pool of cells containing transcriptionally silent but competent proviruses. This is a very tall order, given that we have no specific marker to distinguish the infected from the uninfected cells. Even if some have been proposed, their identification remains very difficult [204,205,206,207,208,209]. An obstacle in assessing the efficacy of cure strategies is the accurate determination of the reservoir size. Measures of proviral DNA in the past have overestimated the viral reservoir size due to the presence of defective proviruses [210,211]. However, current assays are thought to potentially underestimate the reservoir by 60-fold [211]. The “gold-standard” for measuring the replication-competent reservoir has been the quantitative viral outgrowth assay (QVOA), which measures the number of resting CD4^+^ T cells that produce infectious virus after stimulation with an LRA [212]. This might limit quantification since it requires production of not only both viral RNA and protein, but fully assembled virus for detection. The different strategies for detecting infectious virus, proviral DNA, and viral RNA or proteins have been recently reviewed [213]. The actual number of latently infected cells and the real size of the reservoir is unknown [211,214]. Thus, the efficacy of cure strategies remains difficult to assess.

### 7.1. Shock-and-Kill Approach

The most explored approach towards the eradication of latently infected cells is dubbed shock-and-kill. This entails repeated reactivation of latent proviruses using LRAs, in the expectation that reactivated viral products would lead to the eradication of the infected cell by cytopathic effects or elimination by the immune system. Reactivation from latency is performed in the presence of ART to prevent novel infections. Unfortunately, this approach has not been successful so far. Despite some degree of viral RNA production in patients treated with LRAs in clinical trials, there has been no observed reduction in reservoir size [111,112,113,114,115,215]. This could be attributed in part to (1) immune dysfunction and exhaustion of CD8+T cells in HIV-1-infected individuals limiting their ability to clear reactivated cells [216]; (2) the stochastic nature of the reactivation process, not all latent proviruses are activated by a single LRA dose [214,217,218]; (3) toxicity of LRAs on immune cells [219,220]; and (4) many LRAs that were discovered by their ability to reactivate clonal HIV-1-infected cell lines, often failing in the more physiologically relevant models of latency [184]. The latest might be explained by the clonal nature of HIV-1 proviruses in cell lines not being representative of the diversity of proviral sequences and integration sites found in infected individuals. Moreover, differences in the available TFs exist between the resting primary CD4^+^T cells and cell lines, which are continuously proliferating.

Combinations of multiple LRAs, as well as transcriptional modulators might renew the promise of improved “shock” [221]. Multiple transcriptional regulatory factors discussed in this review have been identified as potential targets for HIV-1 reactivation, including the lysine acetyltransferase KAT5 [118], and the methyltransferases CARM1 [108], EZH2, G9a [21], and SYMD2 [120]. Recently, second mitochondria-derived activator of caspases mimetics (SMACm) have come to the forefront in developing cure strategies. SMACms such as Birinapant, GDC-0152, and Embelin have been shown to induce autophagy of HIV-1-infected, but not uninfected CD4^+^ T cells, without inducing viral reactivation [222]. A different SMACm, AZD5582, was shown to increase HIV-1 RNA expression in resting CD4^+^ T cells with limited alterations in cellular gene expression, unlike standard LRAs. These could prove promising as strategies for HIV-1 eradication.

The stochastic nature of the transcription elongation, due to transcriptional circuit fragility [156], influenced by levels of TFs and RNAPII availability, and the chromatin environment of the integration site, influence its ability to be reactivated. Histone deacetylase inhibitors (HDACi) commonly used for latency reversal were shown to have a selective ability to increase the expression of total and elongated HIV-1 transcripts, without a parallel increase in the amount of polyadenylated or multiple-spliced RNAs required for efficient viral production [43]. This might partly explain why clinical trials showed an increase in total viral RNA but no significant reduction in the latent pool. Different LRAs exert different effects on blocks to transcription, suggesting that HIV-1 reactivation induced by LRAs are governed by different mechanisms [43]. In a recent study, the Verdin group assessed the ability of current LRAs to reactivate a novel HIV-1 reporter strain in primary CD4^+^T cells [214]. They found that latency is heterogenous and that none of the LRAs tested reactivated more than 5% of cells carrying latent proviruses. The genomic localization and chromatin context affected the rate of reactivation, in agreement with studies assessing the influence of integration site on transcriptional output [9,214]. In addition to poor efficacy, many LRAs interfere with cellular homeostasis and are cytotoxic to healthy cells, highlighting the need for non-T cell-activating LRAs.

### 7.2. Block-and-Lock Approach

**Figure 6:**
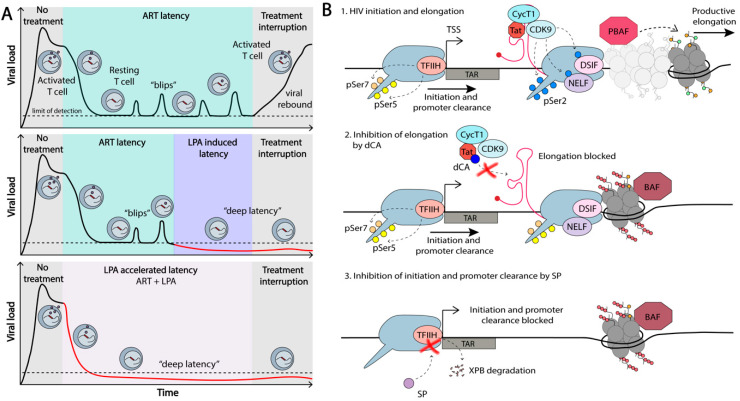
Block-and-lock approach for a functional HIV-1 cure. (**A**) **Top panel**: Upon HIV-1 infection, within a few weeks, there is a peak of viral load (VL) circulating in the plasma of infected individuals. Upon ART initiation, the VL drops to below the limit of detection (50 viral copies/ml), termed “ART latency”. VL “blips,” or episodes of detectable viremia, and are observed in many individuals under ART. Upon discontinuation of ART, there is a rapid rebound of VL to pre-ART levels. **Middle panel**: The addition of a latency promoting agent (LPA), such as the Tat inhibitor dCA, to an ART regimen could suppress the ongoing transcriptional events, induce epigenetic silencing over time, and promote a state of “deep latency”. This might allow for safe ART discontinuation either without viral rebound, or a sufficiently low rebound, such that the immune system is able to control and suppress it. **Bottom panel**: The addition of an LPA at the initiation of ART might accelerate entry into latency, reduce transcriptional events, and potentially limit the size of the reservoir through a reduction in the total number of infected cells. The state of “deep latency” could be achieved earlier, reducing the amount of chronic immune activation in infected individuals and perhaps accelerating the epigenetic silencing of the provirus. (**B**) **1.** When an HIV-1 infected CD4^+^T cell is activated, recruitment of the host TFs and RNAPII allows for efficient PIC formation, initiation of transcription, promoter escape, and some readthrough transcription to produce full length viral transcripts and, thus, Tat. A positive feedback loop is established, where Tat promotes productive elongation through recruitment of P-TEFb and the chromatin remodeler, PBAF. **2.** The Tat inhibitor dCA binds to the basic domain of Tat and blocks Tat interaction with TAR RNA, blocking recruitment of P-TEFb directly to the HIV-1 promoter. Thus, transcriptional elongation is inhibited, and a more repressive chromatin environment can form at the promoter. **3.** The small molecule SP causes degradation of the XPB subunit of TFIIH, which has been shown to inhibit transcription and reduce RNAPII occupancy at both the promoter as well as along the entire genome. SP provides another tool to explore the block-and-lock approach for a functional cure.

In light of the limited success of the shock-and-kill strategy for HIV-1 eradication, an alternative approach was proposed, based on the transcriptional/epigenetic silencing of the virus. This functional cure approach has been termed the block-and-lock approach and aims at a durable viral suppression in the absence of ART [86,223,224]. Much of the human genome is silenced in differentiated cells, e.g., of the ~20,000 genes present in each cell, only approximately 8,000 are transcribed in different cell types [225]. Since it takes energy to activate transcription, it is in a general manner easier to shut down, rather than activate transcription. As such, silencing approaches are increasingly becoming the focus of functional cure approaches [4,5,226]. For success, we need efficient HIV-1 transcriptional silencing agents to “block” and maintain “locked” this silenced state, upon treatment interruption [226] (Figure 6).

The most obvious target for HIV-1 transcriptional inhibition is the viral protein Tat. Any small molecule that inhibits Tat binding to P-TEFb, or Tat-P-TEFb complex binding to TAR, would block transcriptional elongation of the virus. The proof-of-concept for this approach was shown by our group using the small molecule Tat inhibitor, didehydro-cortistatin A (dCA) [227]. In latency cell line models and primary CD4^+^T cells explanted from virally suppressed individuals, dCA potently suppressed viral transcription and reactivation from latency upon stimulation with a series of LRAs (Figure 6) [223,228]. This suppression was sustained even upon removal of ART and dCA. Additionally, dCA reduced viral RNA in tissues, and significantly delayed and diminished viral rebound upon treatment interruption in the bone marrow-liver-thymus (BLT) mouse model for HIV-1 latency [223]. dCA binds the basic domain of Tat, blocking the Tat-TAR interaction and inhibiting Tat-P-TEFb mediated viral transactivation [139]. Long-term treatment with dCA was shown to be associated with a tighter chromatin environment and a loss of RNAPII occupancy on the HIV-1 genome, even under strong stimulating events with LRAs [86]. The classical nucleosome positioning at the LTR was not affected by dCA treatment. Rather, a stronger Nuc/DNA association was promoted, correlating with increased deacetylated H3 at Nuc-1. Additionally, an enrichment of the repressive BAF complex, and a loss of the activating PBAF on the HIV-1 genome was observed. Importantly, this was exclusive to Tat-TAR competent proviruses, excluding off-target activity [86]. Overall, these results highlight the potential of permanently silencing the provirus, which, in addition to preventing rebound, would have the added benefit of reducing some of the symptoms of chronic immune activation observed in ongoing low-grade viral transcription, which might contribute to HIV-1 persistence in ART-privileged sites [229,230]. In summary, dCA is the proof-of-concept that this novel class of molecules that regulate HIV-1 TFs, harbors immense potential in the functional cure for HIV-1.

Other avenues are being explored in the block-and-lock approach for functional cure. HIV-1 appears to be disproportionately sensitive to TNF-α stimulation, as well as to NELF restriction, compared to cellular genes, suggesting that there is a window in which HIV-1 is more sensitive to the suppression of transcriptional elongation than host genes [47]. Since P-TEFb (CDK9/CycT1) is responsible for NELF phosphorylation and the release of proximally paused promoter RNAPII, it is a potential target to be explored in block-and-lock approaches. Inhibition of P-TEFb, for example, with the CDK9 inhibitor flavopiridol, potently blocks viral production [231]. Unfortunately, the therapeutic index of this drug (CC_50_/EC_50_) is around 10, highlighting the crucial nature of P-TEFb for cellular transcription. Furthermore, flavopiridol inhibits other CDKs, including those involved in the cell cycle, and has off-target effects on non-CDK proteins [232]. Nevertheless, strategies targeting transcriptional elongation are a promising avenue to be explored.

Hsp90 inhibitors have also shown potential as HIV-1 transcriptional inhibitors, through reduction of NF-κB-mediated gene expression [233,234]. Hsp90 forms a complex with the chaperone protein Cdc37, and this complex interacts with the NF-κB inhibitor, IKK. Association of IKK with Hsp90/Cdc37 facilitates IκB phosphorylation through stabilization of IKK, which results in the translocation of NFκB to the nucleus to reactivate HIV-1 transcription. Inhibition of Hsp90 has been shown to block this interaction, and thus suppress HIV-1 reactivation [233]. Interestingly, Hsp90 has been implicated in the control of CDK9 levels in cells through the formation of a complex with Cdc37 [95]. Although no changes in P-TEFb concentrations were observed with the short-term inhibition of Hsp90, changes of P-TEFb localization or function on the HIV-1 LTR might explain inhibition of HIV-1 reactivation by Hsp90 inhibitors, and warrants further investigation.

The XPB subunit of the TFIIH complex has been shown to be degraded by the small molecule spironolactone (SP), without obvious global transcriptional defects or cellular toxicity [235,236,237,238,239,240]. Treatment of both cell lines and primary CD4^+^T cells with 10 µM SP was shown to inhibit HIV-1 transcription during acute infection [238]. The lack of toxicity in cells could be explained by work recently published by Frederic Coin’s group [236]. XPB has three enzymatic functions: ATPase, helicase, and DNA translocase [241], and the Coin group has shown that transcription can accommodate a loss of XPB by SP-induced degradation, but not when the ATPase activity of XPB is blocked with Triptolide treatment [236]. The authors argued that the ATPase activity of XPB is necessary for relieving an initiation block that XPB poses itself, and that the cells tolerate a loss of XPB. Our group has recently found that treatment with 10 µM SP of cell line models of latency and primary CD4^+^T cells explanted from ART-suppressed individuals, inhibits HIV-1 transcription, blocks reactivation upon LRA stimulation, and reduces recruitment of RNAPII to the HIV-1 genome (Mori et al., in submission). Thus, XPB presents an interesting target for silencing HIV-1 transcription in functional cure approaches.

The Verdin lab identified mTOR in a genome-wide shRNA screen, as playing a role in HIV-1 latency [242]. Inhibitors of mTOR, such as Torin1, blocked HIV-1 reactivation in CD4^+^ T cells explanted from ART-suppressed individuals. This was concomitant with a reduction in global CDK9 phosphorylation. Inhibition of mTOR blocked both Tat-independent and Tat-dependent reactivation [242], potentially offering additional targets for block-and-lock approaches.

As previously mentioned, BRD4 is known to play an important role in HIV-1 transcription. The BRD4S recruits the repressive BAF chromatin remodeling complex to the HIV-1 promoter, silencing viral expression [90]. BRD4L also competes with Tat for P-TEFb, thus blocking viral elongation. Conversely, BRD4L has also been identified as a positive regulator of HIV-1 transcription. Thus, depending on its interacting partners, BRD4 has versatile roles in HIV-1 transcription, and their modulation could contribute to HIV-1 transcriptional regulation. Interestingly, a recent study involving acute depletion of BET proteins BRD4, BRD3, and BRD2 showed no effect on CDK9 recruitment, but did have global effects on transcriptional elongation [243]. BET proteins were proposed as global regulators of transcription. Additional research is needed to tease out the role of BRD4 in HIV-1 transcription, comparing inhibition of BD domains versus acute depletion of the protein. Specific degraders of BRD4 would be helpful to probe these questions. Recently a selective small molecule was identified that binds to the BD1 domain of BRD4, ZL0580, that inhibits HIV-1 Tat transactivation and elongation [244]. In contrast to the small molecule JQ1, which binds to the BD1 and BD2 domains of all BET proteins and induces viral expression, ZL0580 silences viral transcription via epigenetic suppression in both in vivo and ex vivo models of HIV-1 latency. Treatment of J-Lat cells with ZL0580 cells increased nucleosome occupancy of Nuc-1 on the HIV-1 LTR [244]. A significant delay to rebound upon treatment interruption was observed in cells treated with this compound. It will be interesting to compare the effects of this more specific BRD4 inhibitor with JQ1 treatment.

Finally, the Karn group recently identified the estrogen receptor-1 (ESR-1) as a key regulator of HIV-1 latency [245]. ESR-1 is enriched on latent genomes and exposure to estrogen potently inhibits viral reactivation. This pathway is a pharmacologically attractive target for modulation in therapeutic strategies for functional cure.

The use of the Tat inhibitor dCA to suppress HIV-1 transcription, ultimately epigenetically silencing the HIV-1 promoter, proved the concept of block-and-lock approaches towards a functional cure. Nevertheless, given the Tat-TAR feedback loop and the kinetics of the epigenetic machinery, it is an approach where longer periods of dCA treatment would be needed before the treatment can be interrupted. This would allow a tighter “lock” of the “blocked” promoters. Tat inhibitors are different from other antiretrovirals against HIV-1 in the sense that the longer the treatment, the better the outcome. As with the shock-and-kill approach, it is possible that a combination of targets would be needed to obtain efficient and permanent epigenetic suppression of the HIV-1 promoter. We discussed above the possible cellular factors involved in transcription that could be explored as targets for drug development. However, one of the challenges of targeting host proteins is the risk of off-target activity, again highlighting the potential of targeting viral proteins such as Tat [86]. Screening and lead optimization efforts should thus be placed on the identification of additional small molecules that drive epigenetic silencing of the HIV-1 promoter.

## 8. Concluding Remarks

Efficient production of both full length and multiple-spliced viral RNA involves the collective action of a multitude of host and viral proteins. It is becoming clear that assembly of the transcription machinery on the HIV-1 LTR is not the rate-limiting step for transcription. Instead, it is the efficiency of the transition from PIC assembly and promoter clearance to productive elongation that determines successful viral RNA expression. Advances have been made in recent years towards our understanding of the regulation of these processes, providing novel targets for functional cure strategies.
